# 4C-seq revealed long-range interactions of a functional enhancer at the 8q24 prostate cancer risk locus

**DOI:** 10.1038/srep22462

**Published:** 2016-03-03

**Authors:** Mingyang Cai, Sewoon Kim, Kai Wang, Peggy J. Farnham, Gerhard A. Coetzee, Wange Lu

**Affiliations:** 1 Eli and Edythe Broad Center for Regenerative Medicine and Stem Cell Research, Department of Stem Cell Biology and Regenerative Medicine, University of Southern California, Los Angeles, CA 90033, USA; 2 Zilkha Neurogenetic Institute, University of Southern California, Los Angeles, CA 90033, USA; 3 Division of Biostatistics, Department of Preventive Medicine, University of Southern California, Los Angeles, CA 90033, USA; 4 Department of Psychiatry, University of Southern California, Los Angeles, CA 90033, USA; 5 Norris Comprehensive Cancer Center, University of Southern California, Los Angeles, CA 90033, USA; 6 Department of Biochemistry and Molecular Biology, University of Southern California, Los Angeles, CA 90033, USA; 7 Department of Preventive Medicine, University of Southern California, Los Angeles, CA 90033, USA; 8 Department of Urology, Keck School of Medicine, University of Southern California, Los Angeles, CA 90033, USA

## Abstract

Genome-wide association studies (GWAS) have identified >100 independent susceptibility loci for prostate cancer, including the hot spot at 8q24. However, how genetic variants at this locus confer disease risk hasn’t been fully characterized. Using circularized chromosome conformation capture (4C) coupled with next-generation sequencing and an enhancer at 8q24 as “bait”, we identified genome-wide partners interacting with this enhancer in cell lines LNCaP and C4-2B. These 4C-identified regions are distributed in open nuclear compartments, featuring active histone marks (H3K4me1, H3K4me2 and H3K27Ac). Transcription factors NKX3-1, FOXA1 and AR (androgen receptor) tend to occupy these 4C regions. We identified genes located at the interacting regions, and found them linked to positive regulation of mesenchymal cell proliferation in LNCaP and C4-2B, and several pathways (TGF beta signaling pathway in LNCaP and p53 pathway in C4-2B). Common genes (e.g. *MYC* and *POU5F1B*) were identified in both prostate cancer cell lines. However, each cell line also had exclusive genes (e.g. *ELAC2* and *PTEN* in LNCaP and *BRCA2* and *ZFHX3* in C4-2B). In addition, *BCL-2* identified in C4-2B might contribute to the progression of androgen-refractory prostate cancer. Overall, our work reveals key genes and pathways involved in prostate cancer onset and progression.

Prostate cancer (PCa) is one of the most common types of cancer affecting men worldwide. According to *American Cancer Society: Cancer Facts and Figures 2015*, around 220,800 new cases and 27,540 deaths are expected in the United States in 2015. Genetic predisposition to prostate cancer has been established by epidemiological studies. One of the most important goals of research in the field is to uncover how genetic variations affect phenotype. Genome-wide association studies (GWAS) have been successfully applied to identify genetic variants associated with a specific trait^1^,^2^. Many GWAS explored the association between single nucleotide polymorphisms (SNPs) and diseases, especially ones of a complex genetic nature. GWAS in diverse populations (European ancestry, African ancestry, Japanese ancestry, and Latino ancestry, reviewed in Al Olama *et al*.^3^ and Han *et al*.^4^) have identified >100 independent prostate cancer susceptibility loci and hundreds of potentially functional SNPs^3^,^4^,^5^,^6^,^7^,^8^,^9^,^10^,^11^,^12^,^13^. Of note, the vast majority of these SNPs or their surrogates in linkage disequilibrium are within putative enhancers^12^ in non-coding regions. It was shown that variants in risk enhancers could significantly affect androgen sensitivity in prostate cancer cells^12^. That leads to the question of how these “non-coding” loci alter cell property and confer disease risk. However, limited work has been done to tackle this problem and little is known about the functional mechanism of variants in these non-coding loci.

In this study, we focused on 8q24, an extensively studied locus that has been confirmed as a hotspot increasing prostate cancer risk^14^. Al Olama *et al*.^15^ confirmed three regions at 8q24 as risk regions for prostate cancer — region 1: 128.54–128.62 Mb, region 2: 128.14–128.28 Mb, and region 3: 128.47–128.54 Mb, and also characterized additional linkage disequilibrium blocks. Of note, regions 1 and 3 displayed minimal transcriptional activity^16^, and region 2 showed no association between risk SNPs and the gene expression of nearby transcripts^17^. In addition, cancer risk variants at 8q24 are mostly distributed in a block depleted of genes, with the closest gene being *MYC*, a proto-oncogene that is located ~200 kb telomeric^16^. Thus, we hypothesize that the genetic variation at the 8q24 locus may influence cancer risk by dysregulating the expression of distally located genes. Previous studies suggest that this long-range connection might be possible, often with loci in far-*cis* and even in *trans*. For example, in colorectal and prostate cancer a region encompassing risk variant rs6983267, also located in 8q24, interacts with *MYC* which resides approximately 335 kb telomeric^17^,^18^.

Jia *et al*.^16^ has fully characterized the functional enhancers at the gene-poor 8q24 locus by combining RNA expression, histone modifications and transcription factor binding profiles, and verified their activity using reporter assays. Among the 15 tested enhancer regions called AcP1 through AcP15, AcP10 has the most significant enhancer activities in prostate cancer risk region. Thus we set out to map the genome-wide contacts of the 8q24 risk enhancer AcP10 region in prostate cancer cells, hoping to understand (1) what target genes are influenced by this risk enhancer at a genome-wide scale, and (2) how these interactions contribute to prostate cancer risk. Utilizing circularized chromosome conformation capture (4C) coupled with next-generation sequencing, we can identify all the interacting partners of a given locus of interest at a genome-wide scale^19^,^20^. Previously, our group applied 4C-seq to explore the contact landscapes around the *Oct4* distal enhancer in mouse and human pluripotent stem cells^21^,^22^,^23^. We employed a similar 4C-seq strategy to discover all genomic loci interacting with the enhancer at the 8q24 prostate cancer risk locus.

We selected two representative prostate cancer cell lines: lymph node cancer of the prostate (LNCaP) cells, which were isolated from lymph node metastasis and are androgen sensitive^24^, and C4-2B cells, which were derived from bone metastasis of the LNCaP parental line^25^ and can grow in an androgen independent way, but are still responsive to androgen in certain aspects^26^. We reasoned that the contact landscapes around the 8q24 risk locus revealed by 4C-seq in these two cell lines may shed light on the network(s) regulating prostate cancer initiation and/or progression.

## Results

### We identified 8q24 enhancer interactomes in LNCaP and C4-2B cells

**Figure 1:**
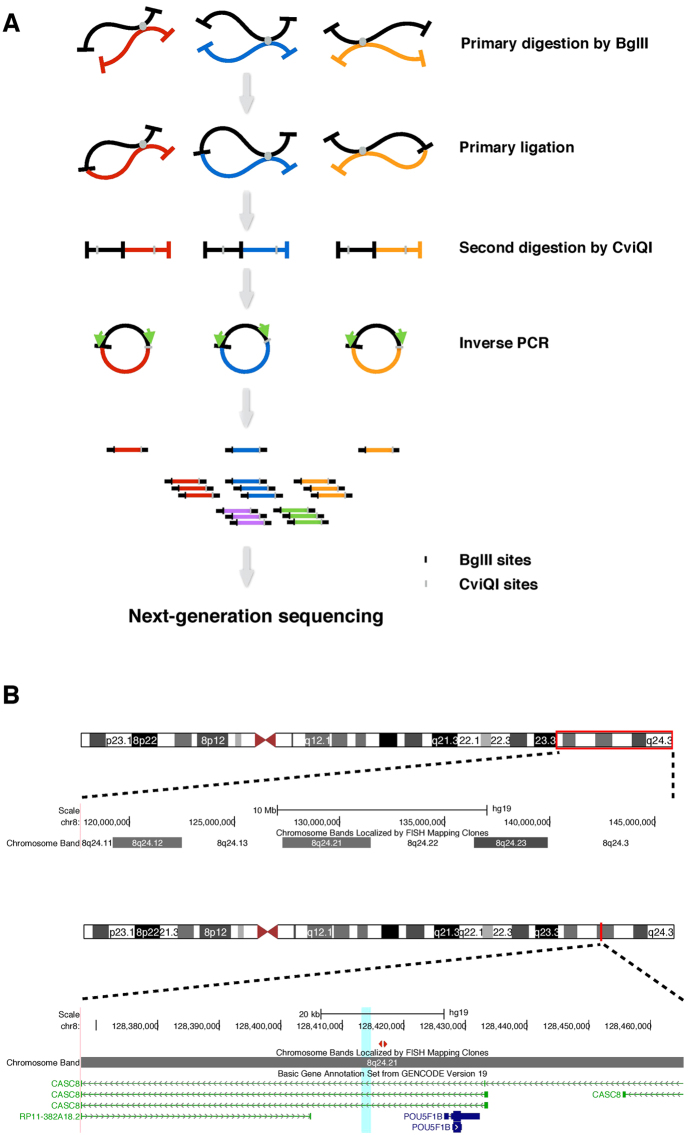
Overview of 4C-seq process and bait region at 8q24 locus. (**A**) “Bait” segment is shown in black while “capture” segments are shown in red, blue and orange. Two rounds of digestion are included, and inverse PCR is performed to amplify unknown captured sequence. (**B**) Upper panel shows a broad view of human 8q24 locus, and lower panel shows a detailed view of AcP10 region at 8q24 locus. AcP10 region is highlighted in cyan. The pair of primers for inverse PCR is shown in red triangles. In gene annotation track, coding gene (*POU5F1B*) is shown in blue, while non-coding genes (*CASC8* and *RP11-382A18.2*) are shown in green.

To identify partners interacting with the 8q24 risk enhancer genome-wide, we applied the 4C technique (Fig. 1A), using enzymatic digestion by BglII as the primary fragmentation method as applied in many other nuclear organization studies^19^,^20^,^21^,^22^. Cells were fixed with paraformaldehyde, and chromosome segments in physical proximity were crosslinked. Then chromatin was digested by BglII, and the ends of interacting chromatin regions were ligated with T4 ligase. We designed primers (Fig. 1B, see Methods) to specifically amplify interacting partners with the bait for library construction. Two replicate libraries for LNCaP and C4-2B were subjected to next-generation sequencing on the Illumina platform. The sequencing reads were analyzed with our custom analysis pipeline^27^. Circos plots depicting the interaction profiles centered on the bait are shown in Supplementary Fig. S1.

**Figure 2:**
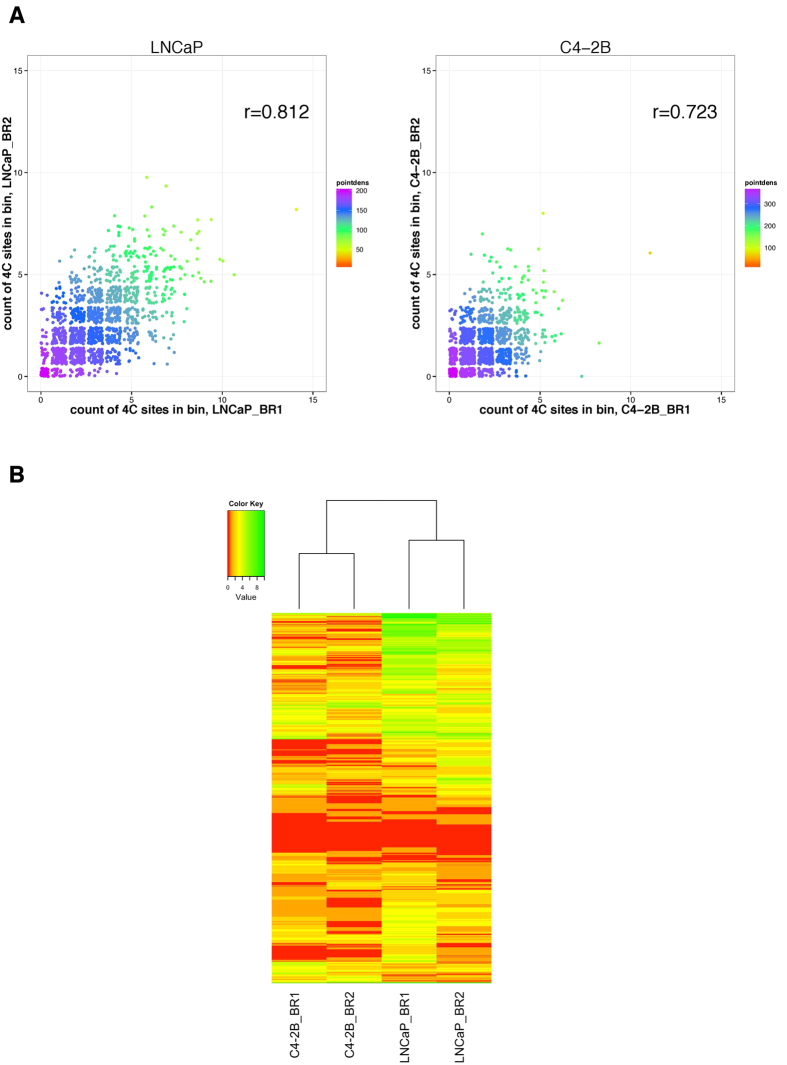
Consistency between replicates. (**A**) Scatter plot showing interactions density. The numbers of interaction sites in each genomic bin (2Mb *trans* and 1Mb *cis*) in two replicates are plotted. Color scale indicates interaction density and Pearson correlation coefficient is shown in the panel. (**B**) Heatmap and dendrogram showing the interactions density and clustering of samples. Complete linkage method with Euclidean distance measure is used. Color scale indicates interaction density. BR: biological replicate.

We evaluated reproducibility of interactions between biological replicates by counting *trans* interactions in every 2 Mb genomic bin and *cis* interactions in every 1 Mb bin. The Pearson’s correlation coefficient was 0.812 between replicates of LNCaP and 0.723 between replicates of C4-2B (Fig. 2A), indicating good consistency between replicates. Furthermore, clustering results showed that replicates of LNCaP and replicates of C4-2B separated well into two clusters (Fig. 2B). This suggests that replicates of the same cell line have consistent 4C results and the risk enhancer interaction profiles of LNCaP and C4-2B differ to some extent.

**Figure 3:**
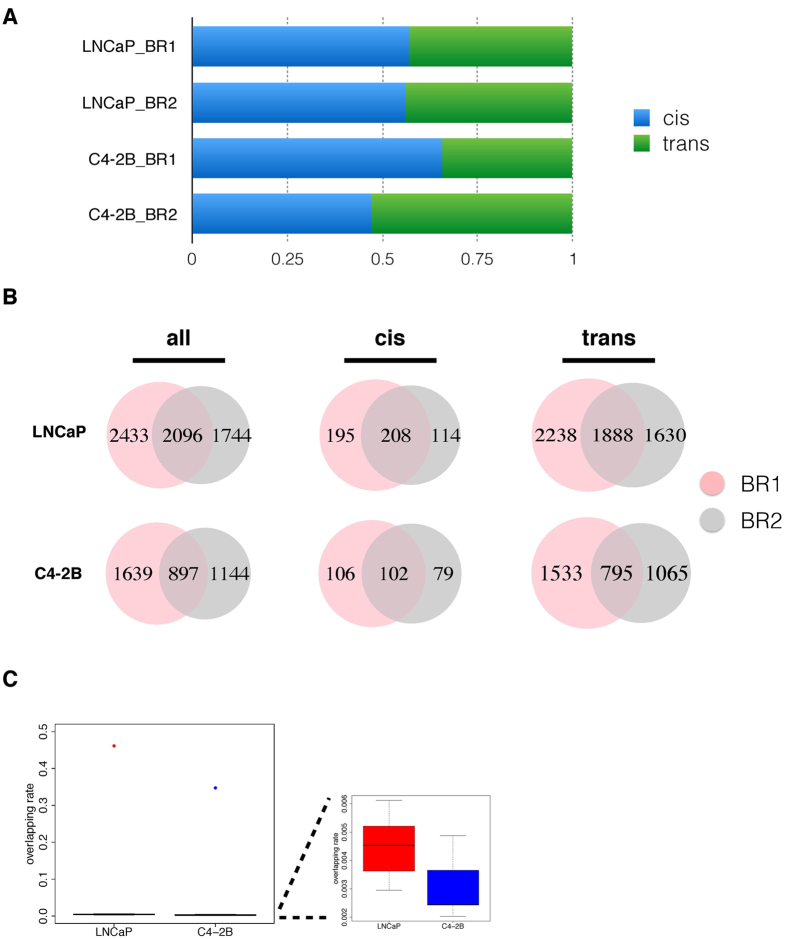
Distribution of 4C reads and sites. (**A**) Stacked bar plot showing the percentage of mapped reads in *cis* and in *trans* in two replicates of LNCaP and C4-2B. (**B**) A Venn diagram showing the number of overlapping sites between two biological replicates in LNCaP and C4-2B. (**C**) Boxplot showing the distributions of overlapping between biological replicates (red dot for LNCaP and blue dot for C4-2B) and between randomly simulated data.

The analysis showed that around 47%~66% of the total reads in four datasets are distributed on the same chromosome where the bait is located (Fig. 3A). This conforms to the “*cis*/overall ratio of >40%” criteria proposed by Van De Werken *et al*.^28^, indicating good quality experiments. After merging reads aligned to the same site, our analysis revealed more than 3,000 interacting sites in LNCaP and more than 2,000 sites in C4-2B (Table 1). Of note, 2,096 sites and 897 sites were reproducibly identified in the two biological replicates of LNCaP and C4-2B, respectively (Table 1, Fig. 3B). Besides, these interactions consist of both *cis* and *trans* sites, with *trans* interactions accounting for the majority (~90%) (Table 1, Fig. 3B). To show that the overlapping between replicates is not by chance, we simulated 10 random sets of equally sized and number of sites for LNCaP and C4-2B respectively, and compared the overlapping rates in true versus random datasets. The median of overlapping rate was 0.45% for LNCaP random sets and 0.24% for C4-2B random sets (Fig. 3C). In comparison, the overlapping rate between biological replicates was much higher, 46.3% and 35.4% for LNCaP and C4-2B respectively, indicating that the overlap observed was not due to chance. In addition, the overlapping rates are comparable to similar studies investigating genome organizations. A previous ChIA-PET study of CTCF-mediated chromatin interactome in pluripotent cells reported the overlapping rate between biological replicates as 38%^29^. We reason that 4C technique takes a snapshot of the chromatin interaction patterns across hundreds of thousands of cells where chromatin-chromatin interactions occur in a highly dynamic fashion. These reproducible sites were thus considered to be high-fidelity interacting sites and would be used to conduct integrative association analysis.

**Figure 4:**
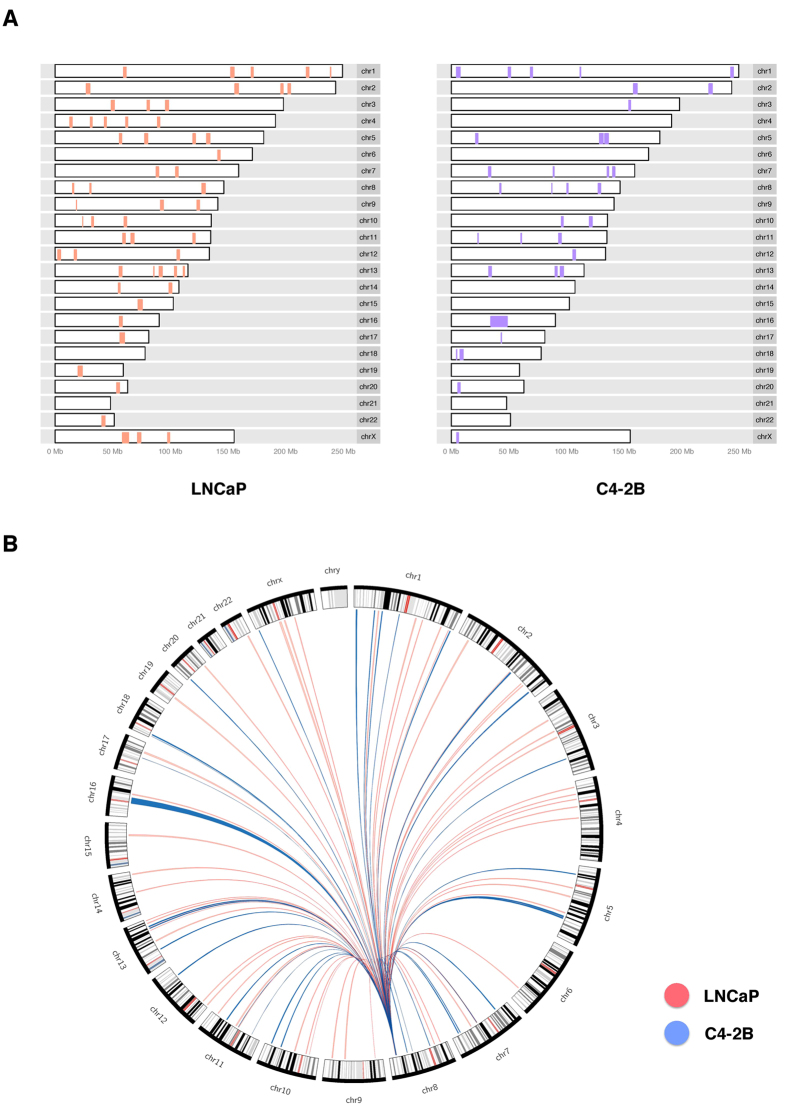
Significant 4C domains. Genome-wide distribution of significant domains in LNCaP (red) and C4-2B (blue) is shown in tabular fashion (**A**) and circos plot (**B**).

We further applied a binomial model based screen to call out significant domains (Fig. 4), and noticed that the distributions of sites interacting with the 8q24 enhancer were very different. This revealed that the chromosome organizations around the enhancer at 8q24 were very different in LNCaP and C4-2B cells. This in turn implies that the enhancer exerts influence over different sets of genes in LNCaP and C4-2B, leading to distinct expression profiles and cancer properties between LNCaP and C4-2B cells.

### The 8q24 enhancer interactomes occupy open chromosomal compartments

Following identification of 8q24 interacting regions, we speculated that the bait-interacting regions could share similar properties. We examined publicly available datasets related to prostate cancer (see Methods) and conducted association analysis following the two-compartment model proposed by Hi-C studies^30^,^31^, where chromosomes are distributed into open compartments enriched with active marks and closed compartments depleted of such marks.

**Figure 5:**
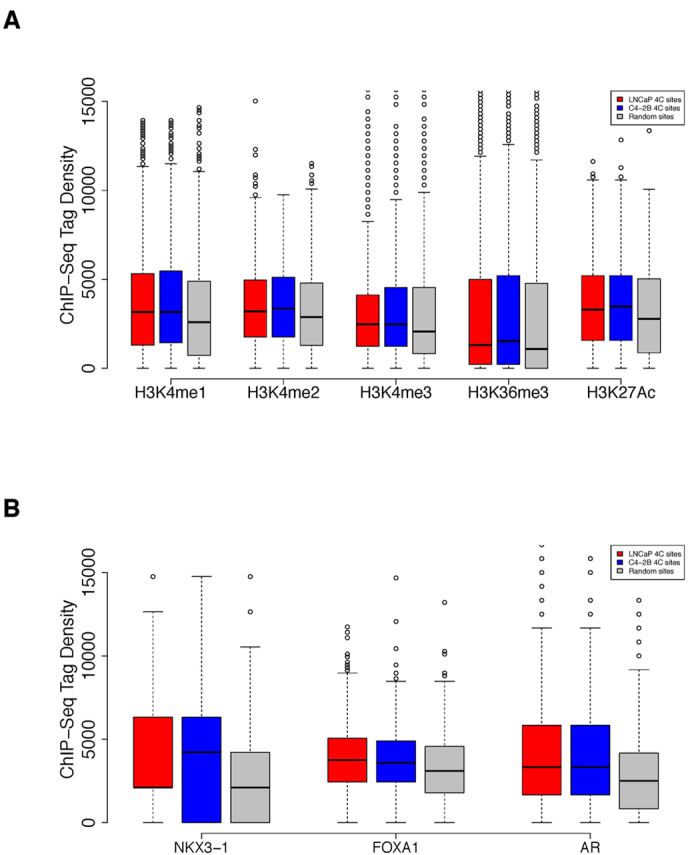
Enrichment of histone marks and transcription factor bindings. (**A**) Boxplot of different open chromatin marks distribution around the interacting sites in LNCaP (red), C4-2B (blue), and random sites (grey). ChIP-seq tags within +/− 500 kb around interacting sites were counted and normalized to 10 million. (**B**) Boxplot of transcription factors binding profile around the interacting sites in LNCaP (red), C4-2B (blue), and random sites (grey). ChIP-seq tags within +/− 500 kb around interacting sites were counted and normalized to 10 million.

Methylation of lysine 4 on Histone H3 — including H3K4me1, H3K4me2 and H3K4me3 — is a hallmark of open chromatin, often featuring promoters and enhancers of actively transcribed genes^32^,^33^. Moreover, these histone modifications facilitate the opening of chromatin by recruiting further chromatin-remodeling factors^34^,^35^,^36^. H3K36me3 is also a mark of active transcription^37^. In addition, H3K27Ac is a reliable and cell-type specific mark of active enhancers and promoters^12^. We examined the enrichment of H3K4 mono-, di- or tri-methylation, H3K36 tri-methylation and H3K27Ac marks around 4C interactome, and found that H3K4me1, H3K4me2, and H3K27Ac were enriched around identified 4C sites (+/− 500 kb) in both LNCaP (H3K4me1: p = 1.088e-6, H3K4me2: p = 7.357e-5, H3K27Ac: p = 5.882e-6; Wilcoxon rank sum test, Fig. 5A) and C4-2B cells (H3K4me1: p = 4.648e-7, H3K4me2: p = 5.435e-5, H3K27Ac: p = 1.903e-6; Wilcoxon rank sum test, Fig. 5A). However, we only observed marginally significant enrichment of H3K4me3 around 4C sites in LNCaP (p = 5.7e-2; Wilcoxon rank sum test, Fig. 5A) and C4-2B (p = 4.24e-2; Wilcoxon rank sum test, Fig. 5A) cells. Besides, H3K36me3 was marginally enriched in LNCaP (p = 2.036e-2) but significantly enriched in C4-2B (p = 6.359e-4). This result suggests that targeted regions of the 8q24 enhancer tend to be transcriptionally active regions marked by H3K4me1, H3K4me2 and H3K27Ac in prostate cancer cells — which could be one of the reasons for their cancerous property.

### The enhancer interactomes are enriched with key transcription factor bindings

Accessible chromatin marked by active histone features is closely related to the occupancy of regulatory factors^38^,^39^. NKX3-1, FOXA1 and AR (androgen receptor)^40^,^41^,^42^ play critical roles in the progression of prostate cancer. We thus compared the ChIP-seq profiles of the three factors and examined their peaks enrichment around our 4C sites (+/−500 kb), revealing that they were significantly enriched around the 4C loci compared to random sites in both LNCaP (NKX3.1: p = 7.214e-7, FOXA1: p = 7.871e-15, AR: p = 5.707e-12; Wilcoxon rank sum test, Fig. 5B) and C4-2B cells (NKX3.1: p = 4.593e-6, FOXA1: p = 6.989e-10, AR: p = 1.786e-11; Wilcoxon rank sum test, Fig. 5B). Therefore, we think these transcription factors can bind to accessible 4C regions and play a critical role in prostate cancer cell function.

### The 4C sites are in close proximity to prostate cancer risk loci

We asked whether 4C sites identified in LNCaP and C4-2B are associated with annotated SNPs in the 100 independently identified prostate cancer risk loci^3^,^5^,^6^,^7^,^8^,^9^,^10^,^11^,^12^. Among the total 112 index-SNPs summarized so far (Supplementary Table S1), 70 and 49 were around +/−1 Mb of 4C sites in LNCaP and C4-2B, respectively. Compared to random sites, 4C sites in both LNCaP and C4-2B are closer to these index-SNPs (p = 6.474e-4 and 7.542e-3 for LNCaP and C4-2B respectively). In particular, rs8014671(14q24: *TTC9*), rs4242382 (8q24.21: *POU5F1B*, *MYC*), rs4242384 (8q24.21: *POU5F1B*, *MYC*), rs7000448 (8q24.21: *LOC727677*, *MYC*), rs1447295 (8q24.21: *POU5F1B*, *MYC*), rs6983267 (8q24.21: *PCAT1*, *MYC*), rs7837688 (8q24.21: *POU5F1B*, *MYC*) and rs188140481 (8q24.21: *PCAT1*, *MYC*) are in close proximity to 4C sites within 10kb in LNCaP. Similarly, rs1447295 (8q24.21: *POU5F1B*, *MYC*), rs4242382 (8q24.21: *POU5F1B*, *MYC*), rs6983267 (8q24.21: *PCAT1*, *MYC*), rs4242384 (8q24.21: *POU5F1B*, *MYC*) and rs7837688 (8q24.21: *POU5F1B*, *MYC*) are within 10kb around 4C sites of C4-2B. We reason that a considerable fraction (>44%) of risk SNPs are around our 4C sites. We think that our 4C pool can facilitate the identification of novel risk loci and risk SNPs, considering that some of the 4C sites are not correlated with those risk loci or SNPs that have been identified by GWAS. Moreover, since we observed the interaction between risk locus 8q24 and other risk loci, we raised the possibility that multiple risk loci could interact with each other forming foci, implicated in cancer onset and development.

### The 4C regions are enriched with pathways and genes involved in prostate cancer activity

**Figure 6:**
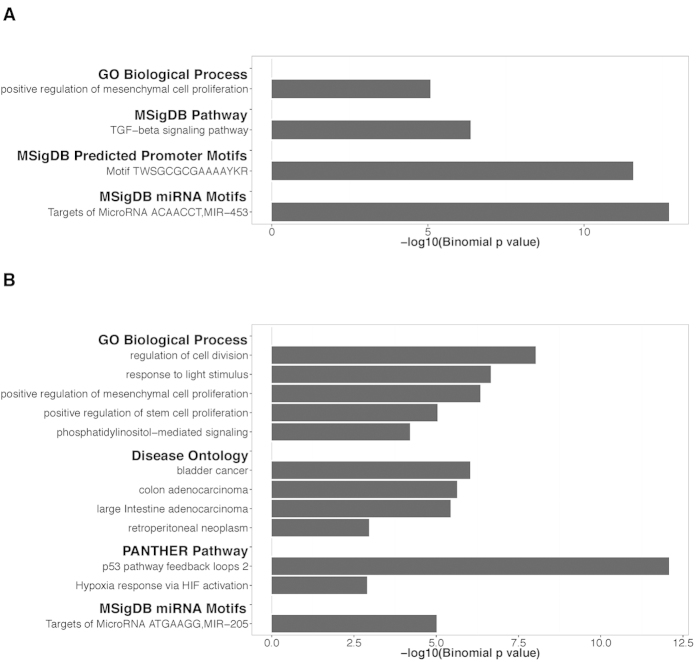
Gene enrichment analysis. Results of gene enrichment analysis in LNCaP (**A**) and C4-2B (**B**).

We input reproducible 4C sites in LNCaP and C4-2B to GREAT^43^ (Genomic Regions Enrichment of Annotations Tool) to analyze functional significance of these sites (Fig. 6, Table 2). We observed significant enrichment of positive regulation of mesenchymal cell proliferation in both LNCaP (p = 8.395e-6) and C4-2B (p = 4.542e-7). A recent study^44^ applying 3C-MTS (chromosome conformation capture based multiple target sequencing) technology to investigate 8q24’s physical interactions with multiple loci across the genome also showed the enrichment of this term. Other terms, including regulation of cell division (p = 9.541e-9), positive regulation of stem cell proliferation (p = 9.131e-6), phosphatidylinositol-mediated signaling (p = 6.312e-5), were found enriched in C4-2B as well. Genes in these terms might be closely involved in prostate cancer activities. In addition, we observed that response to light stimulus was shown as an enriched term in C4-2B, which is interesting considering previous studies proposing melatonin might reduce prostate cancer cell growth^45^ and lower prostate cancer risk^46^. As for pathway analysis, TFG-beta signaling pathway was enriched in LNCaP (p = 4.283e-7), while p53 pathway (p = 8.486e-13) and hypoxia response via HIF activation (p = 1.270e-3) were enriched in C4-2B. These pathways have been demonstrated or proposed as critical in the development of prostate cancer and the regulation of DNA replication, transcription and cell cycle in prostate cancer cells^47^,^48^,^49^,^50^,^51^. The 4C sites were also enriched for some miRNA motifs. For example, MIR-453 (ACAACCT) and MIR-205 (ATGAAGG) were significantly enriched in LNCaP (p = 1.925e-13) and C4-2B (p = 9.812e-6) respectively. Besides, we observed the enrichment of the transcription factor with binding motif TWSGCGCGAAAAYKR in LNCaP (p = 2.699e-12). We found this is an E2F motif in the inverted orientation (TTTSSCGC), implying the role of E2F in prostate cancer development. All the four disease ontology terms (bladder cancer: p = 9.371e-7, colon adenocarcinoma: p = 2.335e-6, large intestine adenocarcinoma: p = 3.691e-6, retroperitoneal neoplasm: p = 1.104e-3) in C4-2B were related to cancer, but no counterparts were found significantly enriched in LNCaP. Of note, under MSigDB Perturbation, the top 4 hits in LNCaP and top 2 hits in C4-2B are all related to cancer (Supplementary Table S2).

Next, we retrieved a set of seed genes associated with prostate cancer from Phenolyzer^52^ (Phenotype Based Gene Analyzer), a tool to discover genes associated with a disease/phenotype term. By comparing GREAT identified genes with the reference genes from Phenolyzer, we found 159 genes in LNCaP and 84 genes in C4-2B related to prostate cancer development (Table 3). 25 prostate cancer related seed genes commonly exist in LNCaP and C4-2B. Among them, *MYC* and *POU5F1B* are the highest ranked genes. Previous studies have shown that irregular chromatin looping and abnormal expression at the *MYC* locus could be a critical, oncogenic driving factor in prostate cancer^53^,^54^. Meanwhile, we noted some seed genes exclusively exist in LNCaP or C4-2B. The highest ranked genes in LNCaP are *ELAC2* and *PTEN*; in comparison, the counterparts in C4-2B are *BRCA2* and *ZFHX3*. As a comparison, the result of Du *et al*.^44^ strengthens our observation of the *cis*-interaction with *MYC* and *trans*-interaction with *FAM84B* (Table 3). In addition to *MYC* and *FAM84B*, Du *et al*.^44^ also showed *CD96*, *PVT1*, *GSDMC*, *CXorf36*, *RRP12*, *USP14*, *SMIN3* as interaction genes. However, except for *GSDMC* in C4-2B, none of these genes were found in our 4C results for LNCaP or C4-2B. Based on Phenolyzer’s analysis, none of these 7 genes is annotated as seed genes associated with prostate cancer, thus it is possible that these interactions with 8q24 locus are not as important as others.

## Discussion

Prostate cancer is the second leading cause of death for men in the United States. Many studies have identified cancer risk associated loci and pathways involved in prostate cancer onset and development. In this study, we employed an unbiased “one-versus-all” technique – 4C-seq to uncover genome-wide interacting partners of a risk enhancer at the 8q24 prostate cancer risk locus. Using LNCaP and C4-2B cell lines for our 4C-seq analysis, we were able to compare these representative models of androgen sensitive and insensitive prostate cancer. Our analysis provides insight into key regions and genes interacting with the risk loci, contributing to our understanding of prostate cancer progression.

As shown above, LNCaP and C4-2B share similar genes interacting with the risk enhancer at 8q24, suggesting a general signature expression landscape for prostate cancer cells. Meanwhile, we also see specific differences in each cell line, where different genes are affected in the same or different enriched pathways. This is understandable given the fact that C4-2B is an osteotrophic and androgen insensitive prostate cancer cell line, unlike its parental LNCaP cell line. The difference in function relies on different set of genes. For example, *BCL-2* gene was an interacting candidate in C4-2B but not in LNCaP. *BCL-2* is an oncogene encoding inner mitochondrial membrane protein that is anti-apoptotic^55^. It has been shown that the transition to a hormone unresponsive state is accompanied with elevated BCL-2 expression, increased proliferation and decreased apoptosis^56^,^57^,^58^. Besides, there is an inverse relation between androgen receptor and BCL-2 expression^59^, which supports the involvement of *BCL-2* in androgen-insensitive cells. To characterize the different picture of gene expression levels in LNCaP and C4-2B, we think RNA-seq can be conducted, which combined with 4C-seq results, will add to our knowledge of how prostate cancer cells transition into hormone-refractory state.

It came to our attention that some pathways and genes related with pluripotent stem cells are enriched, for instance, pathway—positive regulation of stem cell proliferation, and genes—*MYC*, *POU5F1B*. This implies the involvement of cancer stem cells in prostate cancer. Normal pluripotent stem cells and cancer stem cells are similar in many aspects, and the most striking one might be the ability to perpetuate themselves by self-renewal. Normal stem cells can be the targets of transforming mutations and cancer stem cells can drive the cancerous cells proliferation^60^. In addition, Du *et al*.^44^ and our results showed that positive regulation of mesenchymal cell proliferation was an enriched pathway in both LNCaP and C4-2B. Prostate cancer is one type of carcinoma, developing from epithelial cells that line the surface of glands and ducts^61^. Thus it is compelling to see that positive regulation of mesenchymal cell proliferation is an enriched pathway, given that transformed cells of mesenchymal origin will usually lead to sarcoma made of cancerous bone, cartilage, muscle, vascular, hematopoietic tissues and so on. Despite that, it has been shown that during tumorigenesis concomitant changes occur in cells surrounding epithelial cells as well^62^. These cells, called as stromal cells and composed of endothelial, fibroblastic, smooth muscle and nerve cells, play a supportive role for epithelial layer^61^. Several investigations showed that normal molecular dialogue between embryonic epithelial and mesenchymal cells form the basis of tissue/organ function^63^,^64^,^65^, while the disruption of that homeostasis might confer tumorigenesis. That sets the concept framework for the involvement of mesenchymal cells in prostate cancer.

In addition, we realized that some other cancers (for example, bladder cancer, breast cancer and lung cancer, Table 2, Supplementary Table S2) are shown enriched in our ontology analysis of genes interacting with 8q24. 8q24 was found to harbor common alleles in prostate, breast and colon cancer^14^,^66^,^67^. Besides, breast, prostate, colon and lung cancers are common type of carcinomas derived from epithelial cells. Thus it is probable that the risk locus 8q24 interact with similar genes and have common mechanism underlying the association with cancer.

We observed some miRNA motifs enrichment in 4C sites of LNCaP and C4-2B, implying that miRNAs might be implicated in prostate cancer. Over the years, miRNAs have been shown to play a role in regulating gene expression at post-transcriptional level^68^,^69^,^70^. Moreover, evidence is mounting that miRNAs are associated with various diseases, especially cancer^71^,^72^,^73^. Of note, recently an increasing number of studies are dedicated to taking computational approaches to characterize the association between miRNA and disease^74^,^75^,^76^. Our findings might contribute to the knowledge of miRNA-cancer relationship.

In our analysis, we have obtained a list of potentially biologically significant regions and genes in LNCaP and C4-2B cells. We can confirm interactions between the risk enhancer and candidate genes by performing 3C and fluorescent *in situ* hybridization (FISH). To test the biological importance of the interaction with the risk enhancer, we can apply CRISPR (clustered regularly interspaced short palindromic repeats)-Cas9 genome editing to knock out the enhancer region and compare RNA-seq expression profiles between edited and unmodified prostate cancer cells. Recently, Tak *et al*.^77^ has successfully applied this strategy to examine the effect of deletion of a distal element on transcriptome in colon cancer. The genes proposed by computational analysis and experimental validation will be important targets for drug development and intervention therapy.

## Methods

### Cell culture

LNCaP and C4-2B cells were maintained in RPMI 1640 supplemented with 5% (v/v) FBS (fetal bovine serum). LNCaP was obtained from the American Type Culture Collection (ATCC; Manassas, VA), and C4-2B was obtained from ViroMed Laboratories (Minneapolis, MN).

### 4C library preparation

4C library preparation was performed following previously described protocol^28^,^78^ with modifications. 5 million cells were trypsinized and resuspended as single cells in 0.5 ml RPMI 1640/10% FBS. For crosslinking, 9.5 ml of 2% formaldehyde/10% FBS in PBS was added. After being incubated for 10 minutes, fixation was quenched with 0.6 ml of 2.5 M glycine and cells were lysed in 1 ml lysis buffer 1 (50 mM HEPES-KOH, pH7.5, 140 mM NaCl, 1 mM EDTA, 10% Glycerol, 0.5% NP-40, 0.25% Triton X-100, protease inhibitors) for 10 minutes at 4 °C with rotating. Nuclei were pelleted by centrifugation and washed with lysis buffer 2 (10 mM Tris-HCl, pH8.0, 200 mM NaCl, 1 mM EDTA, protease inhibitors). Pelleted nuclei were resuspended in 0.5 ml of 1.2 fold restriction enzyme buffer and incubated with 0.3% SDS for 1 hour at 37 °C with shaking and further incubated with 2% Triton X-100 for 1 hour. BglII restriction enzyme were added and incubated overnight at 37 °C with shaking. Digestion was stopped after adding 1.6% SDS and incubated at 65 °C for 25 minutes. Samples were then diluted in 6.125 ml 1.15 fold ligation buffer and 100 Weiss unit of T4 DNA ligase was added. Samples were kept at 16 °C for 4 hours and then at 25 °C for 30 minutes. The ligated chromatin was digested by proteinase K and then incubated with RNaseA. Samples were purified by phenol-chloroform extraction and ethanol precipitation. Samples were further digested by CviQI and circularized using T4 DNA ligase. After purification, 8 parallel PCR reactions, each containing 1 ug of circularized DNA, were performed (primers: Index primer for replicate 1 –caagcagaagacggcatacgagatTCAAGTgtgactggagttcagacgtgtgctcttccgatcGGAAGTAGAGTAGCAATTCTTG; Index primer for replicate 2 – caagcagaagacggcatacgagatCTGATCgtgactggagttcagacgtgtgctcttccgatcGGAAGTAGAGTAGCAATTCTTG; Universial primer – aatgatacggcgaccaccgagatctacactctttccctacacgacgctcttccgatctATGGAAATCAAGCAGCAGATCT). The amplicons were extracted by E-gel. The bar-coded DNA libraries were sequenced as 50 bp single-end reads using the Illumina HiSeq2000 platform.

### 4C-seq data analysis

Reads with 5’ end mapped to the forward inverse PCR primer sequence were selected. The rest of the selected reads (including the BglII sites) were mapped to hg19 assembly by Burrows-Wheeler Aligner^79^ (BWA). The ligation sites were determined in that process. To get rid of off-target aligned reads, we applied a second round alignment, where the mapped ligated BglII sites were further mapped to a library that includes the locations of genome-wide BglII sites. Sites with only one read were eliminated to filter out random noise. Reproducible sites were defined as exact match of 4C sites with coverage >1 in both biological replicates.

To call out statistically significant regions, we applied a binomial model^19^,^28^. Every interacting site *i* on chromosome *W* with length *L_W_* was examined within window *w* with length *l_w_*. The number of interacting sites was defined as 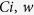, and a *z* score was assigned to the window based on the following calculation:


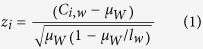


in which *μ_W_* is the expected number of interacting sites in window *w* on chromosome *W* .

A window size of 500 was used in counting the number of ligated sites to define inter-chromosomal interactions, while a window size of 200 was set to define intra-chromosomal interactions. To define intra-chromosomal interactions, we set background window size to 5,000, so as to calculate the expected number of ligated sites in a window. We used type I error rate of 0.05 as the threshold to identify non-random chromosomal interactions.

### Integrative association study

We integrated publicly available datasets that are associated with prostate cancer or LNCaP cells. From the ENCODE Project Portal, we retrieved ChIP-seq data for H3K4me3 (GSM945240). We also retrieved H3K27Ac ChIP-seq data (GSE51621), NKX3-1 ChIP-seq data (GSM699633), FoxA1 ChIP-seq data (GSM699634, GSM699635), and Androgen Receptor ChIP-seq data (GSE28219). The ChIP-seq results for other histone marks were downloaded from the Cistrome database.

### GREAT and Phenolyzer

Genes associated with 4C sites were retrieved by GREAT^43^ (http://bejerano.stanford.edu/great/public/html/), a tool to assign functional annotation to genomic regions. The background regions were chosen as the default whole genome. We used default basal plus extension association rule (proximal: 5 kb upstream, 1 kb downstream; distal: up to 1000 kb). Curated regulatory domains were included.

Seed genes associated with prostate cancer were discovered by Phenolyzer^52^ (http://phenolyzer.usc.edu/), a tool to discover genes based on user inputted disease/phenotype terms. “prostate cancer” was used as input term and a total of 964 genes with ranks were returned.

### Other

Genomic feature annotations were retrieved from the UCSC genome bioinformatics site. Genomic coordinates in other assemblies were converted to GRCh37/hg19 using the *liftOver* tool. Statistical tests and plotting were performed using R.

## Additional Information

**How to cite this article**: Cai, M. *et al*. 4C-seq revealed long-range interactions of a functional enhancer at the 8q24 prostate cancer risk locus. *Sci. Rep*. **6**, 22462; doi: 10.1038/srep22462 (2016).

## Supplementary Material

Supplementary Information

## Figures and Tables

**Table 1:** Summary of metrics in 4C-seq analysis in LNCaP and C4-2B.

cell line	BR	# read pairs	# non-random sites	# overlapping non-random sites	# cis-sites	# overlapping cis-sites	# trans-sites	# overlapping trans-sites
LNCaP	1	27,518,509	4,529	2,096	403 (8.9%)	208 (9.9%)	4,126 (91.1%)	1,888 (90.1%)
	2	38,637,799	3,840		322 (8.4%)		3,518 (91.6%)	
C4-2B	1	25,060,263	2,536	897	208 (8.2%)	102 (11.4%)	2,328 (91.8%)	795 (88.6%)
	2	39,250,858	2,041		181 (8.9%)		1,860 (91.1%)	
Percentages are shown in brackets. Two biological replicates (BRs) were performed for each cell line.								

**Table 2:**
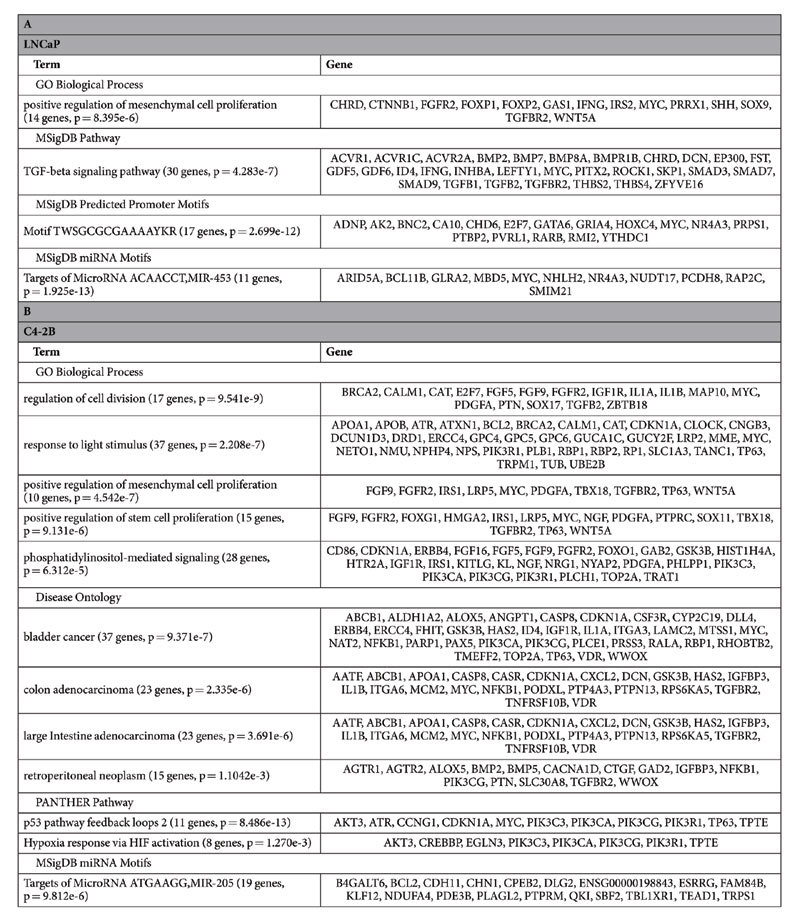
Significant enrichment of ontology annotations of genes associated with 4C sites in LNCaP (A) and C4-2B (B).

**Table 3:** Prostate cancer associated seed genes (annotated by Phenolyzer) identified by 4C-seq in LNCaP and C4-2B.

Cell line	prostate cancer seed genes from Phenolyzer
LNCaP	(159 genes) ELAC2(7), PTEN(10), MSR1(15), EHBP1(16), MAD1L1(22), MYC(30), TNRC6B(38), CTBP2(47), FGF10(49), RAD23B(54), POU5F1B(60), PDLIM5(61), VGLL3(64), PKHD1(68), HAPLN1(69), ANK2(88), CWC22(89), CD180(90), INSC(95), ACTBL2(99), CNTN6(101), TMTC2(103), PTPRU(105), PRPF6(125), ZNF512B(127), KLF4(162), SOX9(163), SIDT1(164), KIAA2018(166), SPICE1(167), BOC(169), SP8(178), ABCB5(179), RAD51B(211), ZFP36L1(212), ATP9B(230), SALL3(234), KRT8(272), DCST2(304), ZBTB7B(308), BOD1(331), STC2(333), PKIA(337), PTGFRN(338), SYK(344), TSN(345), AMIGO2(349), NR2F2(351), INHBA(355), CHL1(358), ZNF608(365), BAI3(366), HEPH(371), GGTLC1(375), FOLH1(381), ERG(385), ESR2(391), PLAU(406), TNFSF10(411), VIM(416), TUSC3(428), CCND1(430), PTGS2(434), TGFB1(437), HIF1A(440), CYP19A1(441), NAT2(446), ESR1(447), CTNNB1(448), MME(450), FHIT(459), TLR4(466), NAT1(467), DAB2IP(479), ADAM9(482), TGFBR2(502), APC(503), GCNT1(507), VAV3(508), MDM2(511), FOXA1(514), CCND2(517), ITPR1(519), BMP7(520), CDH13(526), GJA1(527), GADD45A(528), GHR(531), IL16(539), TPT1(541), BMPR1B(545), PRKACB(550), EIF3H(554), PIK3CD(559), SEPP1(567), PRNP(582), PTPRK(584), FASLG(587), ANXA3(603), PPP3CA(612), APPL2(619), FOXC1(624), GNG5(630), HAO1(631), PCDH8(649), BCAS1(650), CST1(653), TPBG(659), RUNX1(660), DNAJC3(666), ZFP36L2(669), NR3C1(673), PRRX1(674), EMP1(675), PAWR(692), NPR3(708), USO1(710), TLR5(712), SP5(729), TPD52L1(733), NAGK(742), TMEFF2(743), ATAD3A(753), RFK(763), S100P(773), CDYL2(782), SCGB1D1(783), CEP290(786), ID4(788), CHGA(812), PDZD2(813), PKIB(814), SSTR1(823), SV2B(825), LEF1(827), STEAP4(828), TGIF1(829), SLC45A3(836), FST(838), GRP(850), PDC(857), SYT7(861), RCN1(867), ACPP(870), TPCN2(888), FAM84B(891), CBLN1(901), PEX2(902), IRX4(907), PIP4K2A(912), CADM2(915), SLC38A4(921), FSCB(922), GJD2(923), LIG4(948), SGCZ(949), SETBP1(951), SLC10A2(958), KRT78(960)
C4-2B	(84 genes) BRCA2(8), ZFHX3(18), HSD17B3(21), MYC(30), JAZF1(46), FOXP4(51), ANO7(57), POU5F1B(60), ITGA6(65), CWC22(89), PRPF6(125), ZNF512B(127), MMP8(190), TMEM123(194), BIRC2(196), MMP10(197), TANC1(202), CEP57L1(286), ADAR(296), KCNN3(307), KLF11(311), GRHL1(317), FSHR(332), DNAH5(350), NR2F2(351), HEPH(371), APOB(374), GGTLC1(375), VDR(384), ERG(385), IGFBP3(388), UGT2B17(401), BCL2(402), EZR(417), GRPR(418), TUSC3(428), CDKN1A(445), NAT2(446), MME(450), FHIT(459), GSTA1(460), IRS1(481), GSK3B(484), TGFBR2(502), LPAR1(504), FOXA1(514), CYP7B1(518), PODXL(548), ADRB2(551), DAG1(553), SSBP2(556), PIK3CA(557), PARP1(563), ALDH1A2(574), CREBBP(577), ENPP5(617), LIFR(618), GRB7(622), HAO1(631), TOP2A(636), TOM1L1(641), TNFRSF21(645), SLC12A2(665), EMP1(675), MAP3K7(691), PDE4D(730), INADL(736), TMEFF2(743), SPOCK1(765), COX6C(778), ID4(788), TRPV6(792), PSAT1(795), SSTR1(823), FST(838), NOG(842), TCF7L2(843), FAM84B(891), IRX4(907), COL21A1(917), FSCB(922), USH2A(939), SGCZ(949), TES(954)

Numbers enclosed with brackets () are ranks calculated by Phenolyzer. In the table, genes are listed with decreasing rank, and genes exist in both LNCaP and C4-2B results are underlined.
