# Supplementary material for: 4C-seq revealed long-range interactions of a functional enhancer at the 8q24 prostate cancer risk locus
Source: Sci Rep. 2016 Mar 3;6:22462. doi: 10.1038/srep22462 (PMC4776156; doi:10.1038/srep22462)
Supplement: Supplementary Information [file srep22462-s1.pdf]

4C-seq revealed long-range interactions of a functional enhancer at the 8q24 prostate cancer risk locus

Mingyang Cai[1,2,3], Sewoon Kim[1], Kai Wang[2,3,4], Peggy J. Farnham[5,6], Gerhard A. Coetzee[7,8,9], Wange Lu[1,*]

[1]Eli and Edythe Broad Center for Regenerative Medicine and Stem Cell Research, Department of Stem Cell Biology and Regenerative Medicine, [2]Zilkha Neurogenetic Institute, [3]Division of Biostatistics, Department of Preventive Medicine, [4]Department of Psychiatry, [5]Norris Comprehensive Cancer Center, [6]Department of Biochemistry and Molecular Biology, [7]Department of Preventive Medicine, [8]Department of Urology, Keck School of Medicine, University of Southern California, Los Angeles, CA 90033, U.S.A.
[9]Present affiliation: Van Andel Institute, Grand Rapids, MI 49503
[*]Correspondence should be addressed to: wangelu@usc.edu

Supplementary information

Figure S1

Circos plots showing the genome-wide interacting partners with the enhancer bait in two biological replicates (BRs) of LNCaP and C4-2B. Inter-chromosomal interactions (*trans*) are shown in blue, while intra-chromosomal (*cis*) interactions are shown in red.

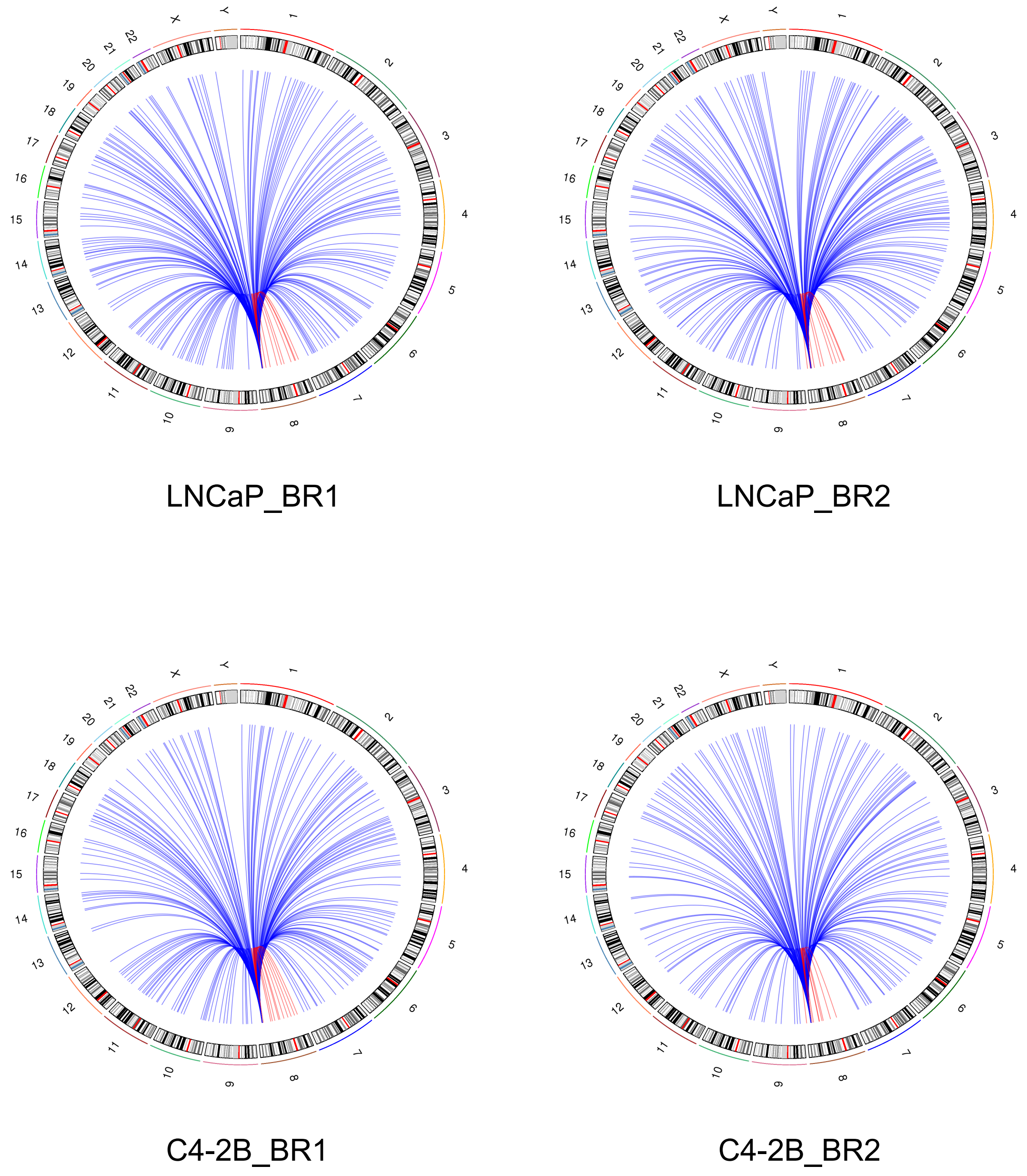

Table S1

Table of independent associations with prostate cancer, revealed by GWAS. Index-SNPs, nearby genes, distance to 4C sites in LNCaP and C4-2B are shown.

#	Loci	SNP	Gene	distance (bp) to 4C sites	
				LNCaP	C42B
**1**	1p35	rs636291	*PEX14*	310044	997082
**2**	1q21	rs17599629	*GOLPH3L*	1711394	837052
**3**	1q32	rs1775148	*SLC41A1*	126163	627348
**4**	1q32.1	rs4245739	*MDM4*	1112814	575268
**5**	2p25	rs9287719	*NOL10*	456039	592563
**6**	2p24.1	rs13385191	*C2orf43*	1394299	155596
**7**	2p21	rs1465618	*THADA*	287592	6140457
**8**	2p15	rs6545977	*EHBP1*	489345	2483152
**9**	2p15	rs721048	*EHBP1, OTX1*	319912	2652585
**10**	2p11.2	rs10187424	*GGCX*	420743	1357183
**11**	2q31.1	rs12621278	*ITGA6*	2075987	281819
**12**	2q37.3	rs2292884	*MPLH*	2497161	3657768
		rs7584330	*MPLH*	2441163	3713766
**13**	3p22.2	rs9311171	*CTDSPL*	1302230	1134408
**14**	3p12.1-2	rs17181170	*CHMP2B*	1028922	7346458
**15**	3p12.1-2	rs2660753	*CHMP2B*	966272	7283808
		rs9284813	*CHMP2B*	1007767	7325303
**16**	3p12.1-2	rs7629490	*CHMP2B*	1097095	7414631
**17**	3q21.3	rs10934853	*GATA2*	955135	504408
**18**	3q23	rs6763931	*ZBTB38*	1398966	1187267
**19**	3q24	rs345013	*PLOD2*	464188	2342232
**20**	3q26.2	rs10936632	*CLDN11, SKIL*	368319	1739541
**21**	4q13	rs10009409	*COX18*	537144	1156742
**22**	4q22.3	rs17021918	*PDLIM5*	239550	1841827
**23**	4q22.3	rs12500426	*PDLIM5*	191282	1890095
**24**	4q24	rs7679673	*TET2*	1597460	2524355
**25**	5p15.33	rs2242652	*TERT*	971319	1375285
**26**	5p15.33	rs12653946	*LPCAT1*	355518	759484
**27**	5p12	rs2121875	*FGF10*	624378	2958808
**28**	5q14.3	rs4466137	*HAPLN1*	464022	2003639
**29**	5q23.1	rs37181	*COMMD10*	313759	544806
**30**	6p24	rs4713266	*NEDD9*	214340	119101
**31**	6p22	rs115457135	*TRIM31*	2725875	4054106
**32**	6p21	rs115306967	*HLA-DRB6*	1334532	3504907
**33**	6p21.1	rs1983891	*FOXP4*	1039928	53652
**34**	6p12.2	rs10498792	*PKHD1*	155463	1037820

**35**	6q14	rs9443189	*MYO6*	2099055	1655131
**36**	6q22.2	rs339331	*RFX6*	188930	3965316
**37**	6q25.3	rs651164	*IGF2R*	1135518	1365600
**38**	6q25.3	rs9364554	*SLC22A3*	883228	1617890
**39**	7p15.3	rs12155172	*RPL23P8*	100986	1126916
**40**	7p15.2	rs10486567	*JAZF1*	497289	248562
**41**	7p12	rs56232506	*TNS3*	221719	1081958
**42**	7q21.3	rs6465657	*LMTK2*	1241326	959941
**43**	8p21.2	rs1512268	*NKX3-1*	222633	629826
**44**	8q24.21	rs12543663	*LOC727677, MYC*	20668	28124
**45**	8q24.21	rs10086908	*POU5F1B, MYC*	42405	25033
**46**	8q24.21	rs1016343	*POU5F1B, MYC*	36460	29396
**47**	8q24.21	rs13252298	*PCAT1, MYC*	34601	31255
**48**	8q24.21	rs1456315	*PCAT1, MYC*	25820	40036
		rs13254738	*PCAT1, MYC*	25414	40442
**49**	8q24.21	rs6983561	*PCAT1, MYC*	22877	42979
**50**	8q24.21	rs188140481	*PCAT1, MYC*	8851	16940
**51**	8q24.21	rs16902094	*PCAT1, MYC*	15931	13759
**52**	8q24.21	rs445114	*PCAT1, MYC*	13096	10924
**53**	8q24.21	rs6983267	*PCAT1, MYC*	3497	3497
**54**	8q24.21	rs7000448	*LOC727677, MYC*	2361	14526
**55**	8q24.21	rs1447295	*POU5F1B, MYC*	2766	1044
		rs4242382	*POU5F1B, MYC*	185	3021
		rs4242384	*POU5F1B, MYC*	1166	4002
		rs7837688	*POU5F1B, MYC*	8771	8771
**56**	9p21	rs17694493	*CDKN2B-AS1*	799273	657023
**57**	9q31.2	rs817826	*KLF4*	39701	3869987
**58**	9q33.2	rs1571801	*DAB2IP*	237755	1871944
**59**	10q11	rs76934034	*MARCH8*	331021	187871
**60**	10q11.23	rs10993994	*NCOA4*	1170352	5654380
		rs3123078	*NCOA4*	1145827	5629855
**61**	10q26.12	rs11199874	*FGFR2*	62928	139096
**62**	10q26.13	rs4962416	*CTBP2*	313181	2092329
**63**	11p15.5	rs7127900	*IGF2*	1468702	982779
**64**	11q13.2	rs10896449	*CCND1*	327389	810062
		rs7931342	*CCND1*	327559	809892
**65**	11q13.2	rs12418451	*CCND1*	386637	750814

**66**	11q13.2	rs11228565	*CCND1*	343476	793975
		rs7130881	*CCND1*	326098	811353
**67**	11q23	rs11214775	*HTR3B*	810046	1596593
**68**	12q13	rs80130819	*RP1-228P16.4*	402683	137657
**69**	12q13.12	rs731236	*VDR*	221822	43199
**70**	12q13.13	rs10875943	*TUBA1C*	1368181	1394049
**71**	12q13.2	rs902774	*KRT8*	22319	1737763
**72**	12q21.31	rs12827748	*PAWR*	614987	1877260
**73**	13q22.1	rs9600079	*KLF5*	230010	1821369
**74**	13q33.2	rs1529276	*MIR548AS*	120103	1555014
**75**	14q23	rs7153648	*SIX1*	964273	3182251
**76**	14q24	rs8014671	*TTC9*	75	70518
**77**	15q21.1	rs4775302	*SQRDL*	1417417	1935283
**78**	16q22	rs12051443	*PHLPP2*	2601204	1394237
**79**	17p13.3	rs684232	*VPS53*	4597918	11369793
**80**	17q21.2	rs7501939	*HNF1B*	2150905	491126
		rs4430796	*HNF1B*	2147789	488010
**81**	17q21.2	rs11649743	*HNF1B*	2124728	464949
**82**	17q21.33	rs138213197	*HOXB13*	1589507	1354511
**83**	17q21.33	rs11650494	*ZNF652*	2128988	815030
**84**	17q21.33	rs7210100	*ZNF652*	2186070	723467
**85**	17q25.1	rs1859962	*BC039327*	114025	1856982
**86**	19q13.4	rs103294	*LILRA3*	101827	2972421
**87**	19q13.11	rs8102476	*SPINT2*	3055381	13089809
**88**	19q13.12	rs887391	*LOC100505495*	194625	9839798
**89**	19q13.32	rs2735839	*KLK3*	1614289	460799
**90**	20q13	rs12480328	*ADNP*	47254	1181529
**91**	21q22	rs1041449	*TMPRSS2*	478838	2591338
**92**	22q11	rs2238776	*TBX1*	3346645	1811536
**93**	22q13.1	rs9623117	*TNRC6B*	216549	1830151
**94**	22q13.2	rs742134	*PACSIN2*	1825421	1236000
**95**	22q13.2	rs5759167	*BIK*	1807358	1217937
**96**	Xp11	rs2807031	*XAGE3*	1780960	9017210
**97**	Xp11.22	rs5945572	*NUDT11*	3448226	10684476
		rs5945619	*NUDT11*	3436237	10672487
		rs1327301	*NUDT11*	3467852	10704102
**98**	Xq12	rs5919432	*AR*	699867	1560291
**99**	Xq13	rs6625711	*SLC7A*	1752304	1277068
**100**	Xq13	rs4844289	*NLGN3-BCYRN1*	1484171	1008935

Table S2

Enriched MSigDB Perturbation terms related with cancer and reported by GREAT. The results of LNCaP and C4-2B are shown in (A) and (B) respectively.

A

**LNCaP**		
**MSigDB Perturbation terms**	**p-value**	**genes**
Genes bound by ESR1 [GeneID=2099] and down-regulated by estradiol [PubChemID=5757] in MCF-7 cells (breast cancer) expressing constitutevly active form of AKT1 [GeneID=207]	3.266e-11	CDYL2, CPEB4, DLL1, EDN2, ESR1, FAM84B, GABBR2, GPD2, IL1R1, LMBRD1, MKX, NPAS3, PPAP2B, PSD3, S1PR3, SEMA3E, SHROOM3, ST8SIA4, SYTL2, TGFB2, TLE1, UBE2H, YPEL2, ZDHHC7, ZMYND8
Genes up-regulated in A549 cells (lung cancer) expressing MIRLET7A3 [GeneID=406883] microRNA off a plasmid vector	6.439e-11	ADCK2, ARHGAP26, BCHE, BTG1, CHGB, COX7C, CPEB4, CTH, DKK1, EHBP1, ETNK1, FAM84B, FAT1, FGL1, FOXA1, FOXC1, GJA1, GLIS3, GLRX, KLF4, LYST, MAGEA6, PLA2G4A, PTPN12, REG3G, SLC38A2, TFPI, THSD4, THSD7A, USP32
Genes present but differentially expressed between Hep3B cells (hepatocellular carcinoma, HCC) with RNAi knockdown of SDHB [GeneID=6390] and control cells	1.390e-9	A2M, AGTR1, CDH6, CTBP2, CYB561, FAM84B, FLRT2, GJA1, GPRC5B, GULP1, HDAC9, HS3ST3B1, ISX, ITPR2, MICAL2, NUAK1, PAG1, PKHD1, PRRX1, SERPINE2, SH3BGRL, SIPA1L2, STEAP1, TFAP2A, TGFB2, THBS4, TNFRSF19, TNFSF4, TTR, VGLL3
Genes bound by ESR1 [GeneID=2099] and down-regulated by estradiol [PubChemID=5757] in MCF-7 cells (breast cancer)	2.602e-9	ANK3, CDYL2, CHRM3, CPEB4, DLL1, EDN2, ESR1, EXT1, FAM84B, FRMD6, GATA3, KCTD18, NPAS3, PBX1, PSD3, PTPRK, RERE, S1PR3, SEMA3E, SHROOM3, SLITRK6, SMAD3, SYTL2, TGFB2, TLE1, TNFRSF19, TNS3, TRAM2, UBE2H, YPEL2

B

**C4-2B**		
**MSigDB Perturbation terms**	**p-value**	**genes**
Genes within amplicon 8q23-q24 identified in a copy number alterations study of 191 breast tumor samples	2.261e-27	COL14A1, ENPP2, FAM84B, FER1L6, GML, HAS2, LY6D, MAL2, MED30, MRPL13, MTSS1, MYC, NOV, PTP4A3, RAD21, SLC30A8, SLURP1, THEM6, TMEM65, TMEM74, TRIB1, TRPS1, TSNARE1, UTP23, ZHX2, ZNF572
Genes bound by ESR1 [GeneID=2099] and down-regulated by estradiol [PubChemID=5757] in MCF-7 cells (breast cancer) expressing constitutevly active form of AKT1 [GeneID=207]	2.638e-11	ARID5B, CGN, EPS8, FAM84B, GABBR2, GALNT10, GRHL1, KLF9, MKX, NPAS3, PPAP2B, PSD3, SLC22A23, TGFB2, ZNF277
